# CNN-based dual-morphology mosquito recognition and mobile deployment

**DOI:** 10.1371/journal.pone.0359976

**Published:** 2026-10-09

**Authors:** Lei Zhu, Jiahao Duan, Jing Wang, Deming Ma, Ming Kong, Lu Liu, Liangliang Huo

**Affiliations:** 1 Institute of Infectious Disease Control and Prevention, Hangzhou Center for Disease Control and Prevention (Hangzhou Health Inspection Institution), Hangzhou, China; 2 College of Metrology Measurement and Instrument, China Jiliang University, Hangzhou, China; Institut National de la Recherche Agronomique, MOROCCO

## Abstract

Mosquito-borne diseases pose a severe global public health threat. Accurate and rapid identification of mosquito species, sex, and developmental stages is critical for effective vector control and disease prevention.Traditional morphological identification is time-consuming, labor-intensive, and highly dependent on expert experience.To address these limitations, we developed a deep learning-based mosquito detection system that integrates adult and larval morphology for recognition and can be deployed on mobile devices.We constructed a dual-morphology mosquito dataset containing 2,683 images, covering three major disease-transmitting genera (*Aedes*, *Culex*, *Anopheles*), including adult males and females, as well as *Aedes* and *Culex* larvae. We compared five object detection algorithms: YOLOv8, YOLOv5, SSD, Faster R-CNN. The results showed that YOLOv8 achieved the best comprehensive performance, with precision = 0.988, recall = 0.990, mAP50 = 0.992, F1-score = 0.99, and only 2.7 M parameters, enabling fast inference suitable for mobile deployment.We further implemented the optimized YOLOv8 model on Android mobile platforms using the NCNN framework. The system supports both static image recognition and real-time video detection with high accuracy. This study provides a portable, automated, and practical tool for on-site mosquito surveillance, which can help improve the efficiency and accuracy of vector-borne disease control programs.

## 1. Introduction

Mosquito-borne diseases constitute a severe global public health burden, with over 265 mosquito-isolated viral species accounting for nearly half of all known vector-borne viruses [1]. These diseases are highly endemic in multiple regions worldwide, including Southeast Asia, Africa, China, and South America. Malaria, filariasis, dengue, and Japanese encephalitis (JE) prevail in many affected regions, while yellow fever and West Nile fever circulate in other epidemic areas [2]. As the deadliest vector animals worldwide, mosquitoes exhibit strict species-specific pathogen transmission. *Aedes* mosquitoes transmit dengue and Zika viruses [3], *Culex* serves as the primary vector for JE [4,5], and *Culex* and *Anopheles* sinensis jointly transmit lymphatic filariasis pathogens [6]. Dominant in China’s mosquito fauna [7], *Culex* and *Aedes* are core surveillance targets, rendering their precise monitoring critical for blocking disease transmission [8].

Only female mosquitoes transmit pathogens via blood meals, and larval stage identification enables effective early intervention—larval nutrition even impacts adult viral susceptibility and transmission [9–11]. Viral vertical transmission in mosquitoes [12] further underscores the urgency of automatic recognition of mosquito sex and developmental stages. Hence, automated, accurate identification of mosquito developmental stages and sex is essential for early vector intervention. Traditional mosquito surveillance relies on manual microscopic identification, which is labor-intensive, time-consuming, and susceptible to human subjectivity and environmental interference [13]. It also imposes infection risks on operators and entails high equipment costs. Aggravated by the global decline in professional mosquito taxonomy expertise [14–16], conventional methods fail to meet large-scale, high-efficiency surveillance demands.

Deep learning, particularly convolutional neural networks (CNNs), has revolutionized intelligent vector detection. Classic CNN architectures including AlexNet [17], GoogleNet [18], VGGNet [19], ResNet [20], DenseNets [21], and MobileNet [22] have been widely adopted for mosquito species classification, achieving recognition accuracy exceeding 95% in prevailing studies [23–28]. Furthermore, CNN-based object detection algorithms (YOLO, Faster R-CNN, SSD) enable integrated localization and classification of multi-category targets in complex field scenarios [29–31]. Recent studies have verified the efficacy of these frameworks in mosquito detection, with optimized models achieving up to 99% detection accuracy, demonstrating great application potential for vector monitoring [13,32,33].

Nevertheless, existing research focuses overwhelmingly on adult mosquito species identification, while studies on sex classification and larval morphological detection remain insufficient. Given that larval source reduction is more sustainable and efficient than adult mosquito control, this morphological imbalance creates a critical research gap in refined vector surveillance. To address this issue, this study constructs a dual-morphology mosquito dataset covering *Aedes*, *Culex* and *Anopheles* with adult/larval morphological and sex annotations. Five mainstream object detection models were compared, and the lightweight, high-precision YOLOv8 model was optimized for multi-dimensional mosquito identification. The trained model was further deployed on Android mobile devices for field real-time detection. This work provides a portable, reliable intelligent tool for on-site vector monitoring, facilitating the automation and refinement of mosquito-borne disease prevention and control. The study flow chart is shown in Fig 1.

**Fig 1:**
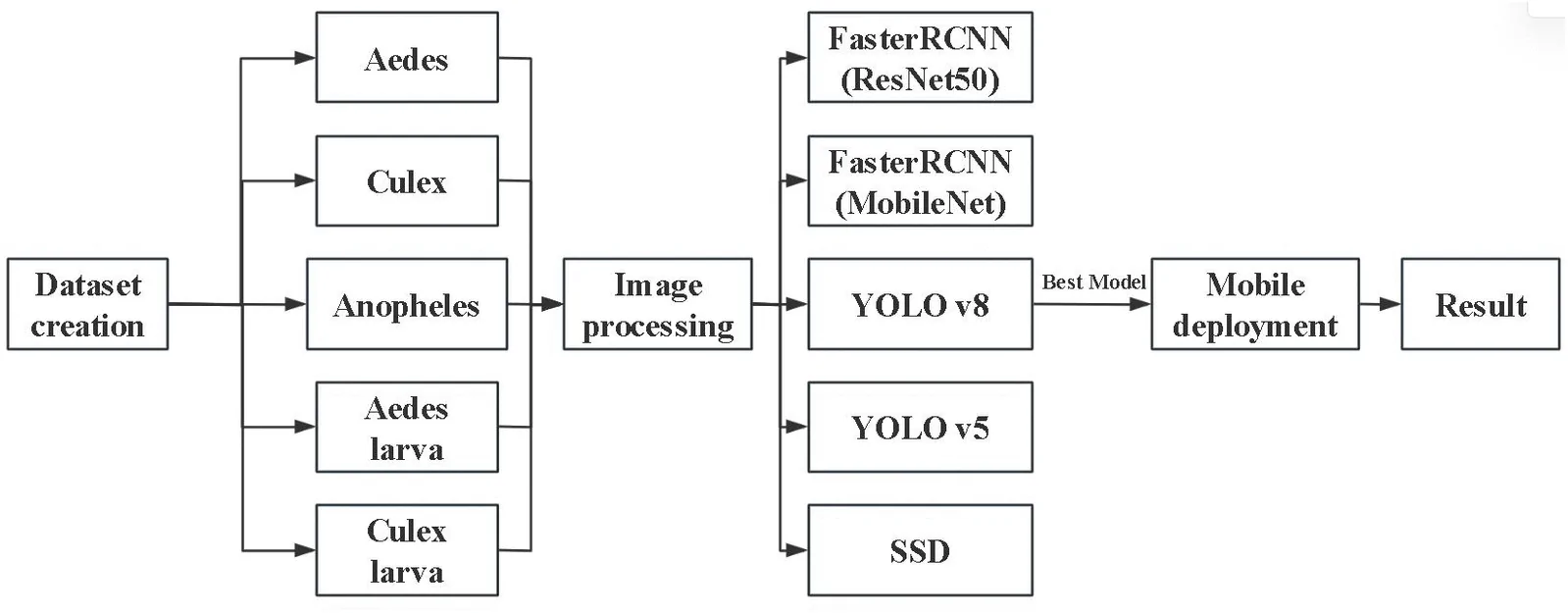
Study flowchart.

## 2. Materials and methods

### 2.1. Dataset preparation

The dataset used in this study consists of 2,683 images, with mosquito samples obtained from three sources: field-collected specimens, laboratory-reared strains purchased from Siweishengwu Biotechnology Co., Ltd., a professional mosquito breeding facility, and publicly available images from the Kaggle mosquito dataset. Fig 2 shows the equipment used for image acquisition in this study. Fig 3a presents the dataset structure, showing the instance count for each mosquito category. The labels are organized based on biological taxonomy and characteristics, including species-level classes (*Aedes*, *Culex*, *Anopheles*), sex-based labels (male and female), and developmental stages (*Aedes* larva and *Culex* larva). Sample images for each category are displayed, illustrating the morphological differences between species, sexes, and larval stages, as shown in Fig 3b. The obtained mosquito samples were photographed using a Supereyes digital microscope equipped with a KSGAOPIN LED ring light source, ensuring consistent and uniform illumination for all specimens. A standardized plastic specimen measuring plate was used as the background, with blue and white being the background colors. To simulate practical conditions for mobile deployment, a subset of mosquito images was captured using Android smartphones and incorporated into the dataset. To prevent the model from overfitting to background features, approximately 10% of the images were taken against blank backgrounds to enhance generalization. All images were captured from a top-down perspective to maintain consistency in shooting angle and specimen orientation.

**Fig 2:**
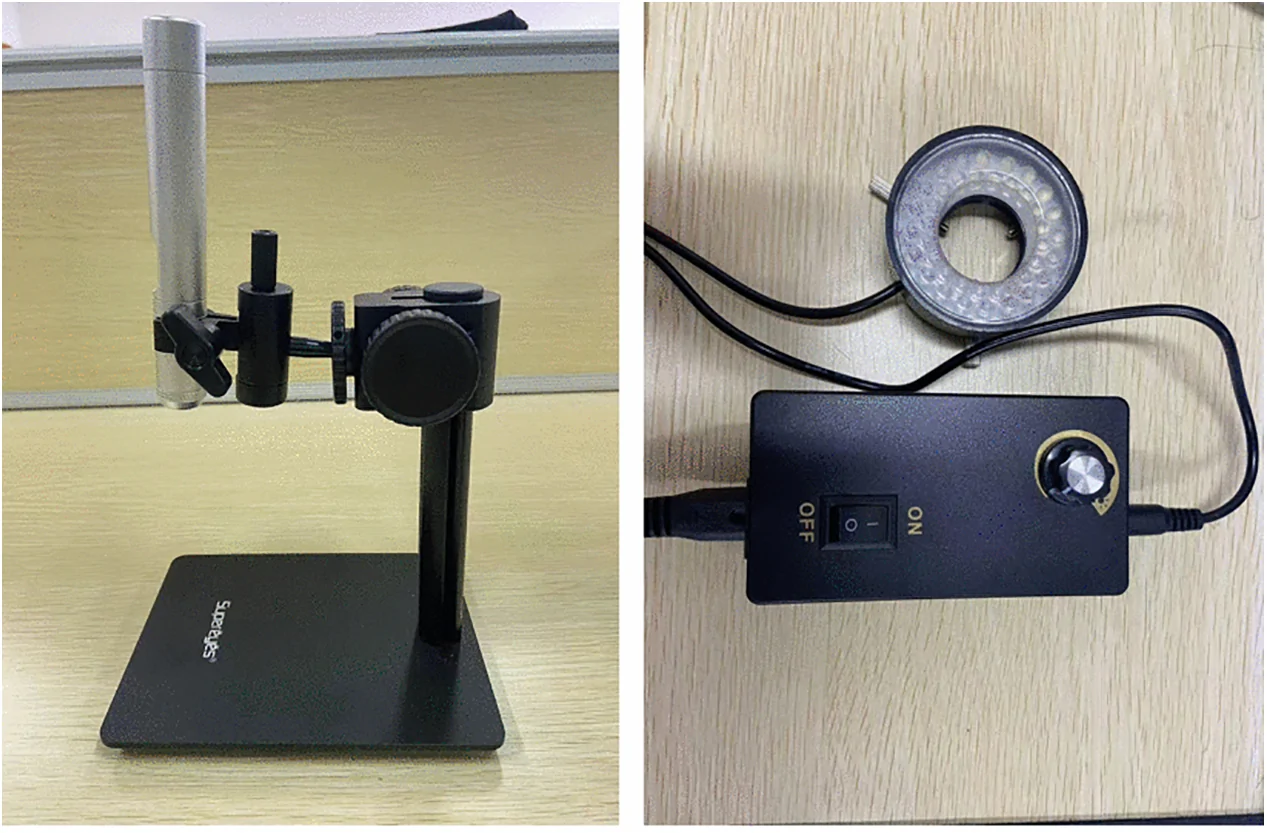
Equipment used for image acquisition in this study. (a) Supereyes digital microscope. (**b**) LED ring light source.

**Fig 3:**
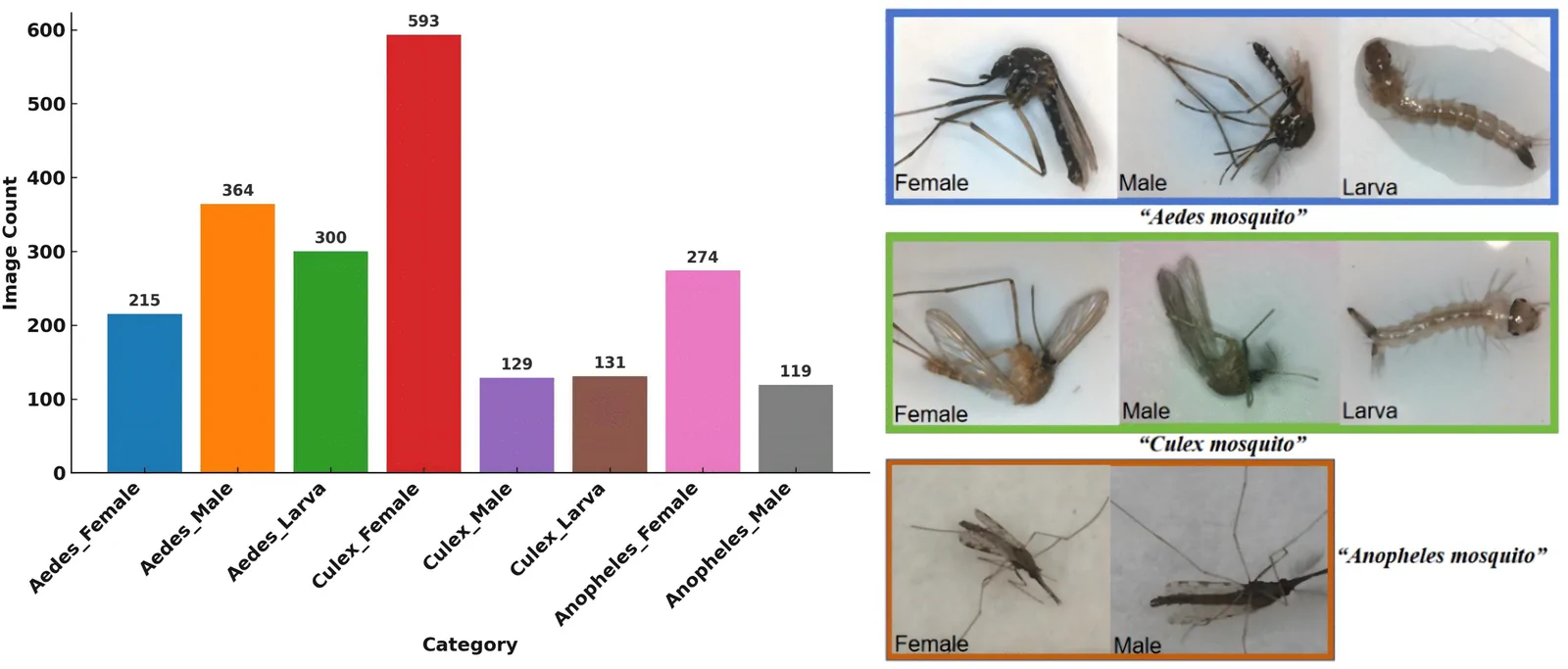
Dataset Distribution and Sample Images of Mosquito Categories. (**a**) Dateset Structure. (**b**) Sample Categories.

The images collected for the study were annotated using LabelImg software, with annotations based on the biological characteristics of the mosquitoes, as outlined in Table 1. Once the images were captured and annotated, the dataset was randomly split into training, validation, and test subsets, following a ratio of 8:1:1. This partitioning was selected to allocate sufficient data for model training while retaining independent validation and test subsets for model selection and final performance evaluation, respectively. Data augmentation was applied only to the training set after dataset partitioning to avoid introducing augmented samples into the validation and test sets. As depicted in the category distribution (Fig 3a), the dataset shows an imbalance between classes, especially for the minority categories such as *Culex*-larva, *Aedes*-larva, and male mosquitoes.

**Table 1:** Morphological features of mosquitoes from three medically important genera.

Mosquito Species	Adult Sex Differences	Larval Morphology	Sex Differences
*Aedes*	Black body with distinct white scale markings on legs and thorax	Short, thick siphon with low siphon index and few pecten teeth; wide and rounded head capsule	Male: strongly plumose antennae and long maxillary palps; Female: pilose antennae and short palps
*Culex*	Light brown body with no distinct markings; blunt abdomen	Long, slender siphon with high siphon index and multiple evenly spaced pecten teeth; narrow head capsule	Male: moderately plumose antennae and elongated palps; Female: sparsely pilose antennae and shorter palps
*Anopheles*	Slender gray body with dark wing spots; palps as long as proboscis	No siphon: larvae float parallel to water surface; rounded head capsule	Male: densely plumose antennae and clubbed palps; female: less plumose antennae and straight palps

To address this issue, various image-level augmentation techniques--such as horizontal flipping, random rotation, translation, scaling, and brightness adjustments--were employed to increase the representation of these underrepresented categories. The augmented samples were added to the training set, which not only helped balance the classes but also enhanced the model's ability to generalize to minority classes. The updated sample distribution after augmentation is provided in Table 2.

**Table 2:** Sample counts after data augmentation.

Categories	Original Count	Augmented Count
*Aedes*_Female	215	577
*Aedes*_Male	364	600
*Aedes*_Larva	300	607
*Culex*_Female	593	650
*Culex*_Male	129	549
*Culex*_Larva	131	572
*Anopheles*_Female	274	424
*Anopheles*_Male	119	427
Background	440 (≈10% of the total images)	

### 2.2. Model selection

For real-time mosquito detection on mobile devices, a lightweight model is ideal, and YOLOv8 (Ultralytics)—a mainstream variant of the YOLO series—was thus selected for this study [34]. Fig 4 shows the workflow of YOLOv8.

**Fig 4:**
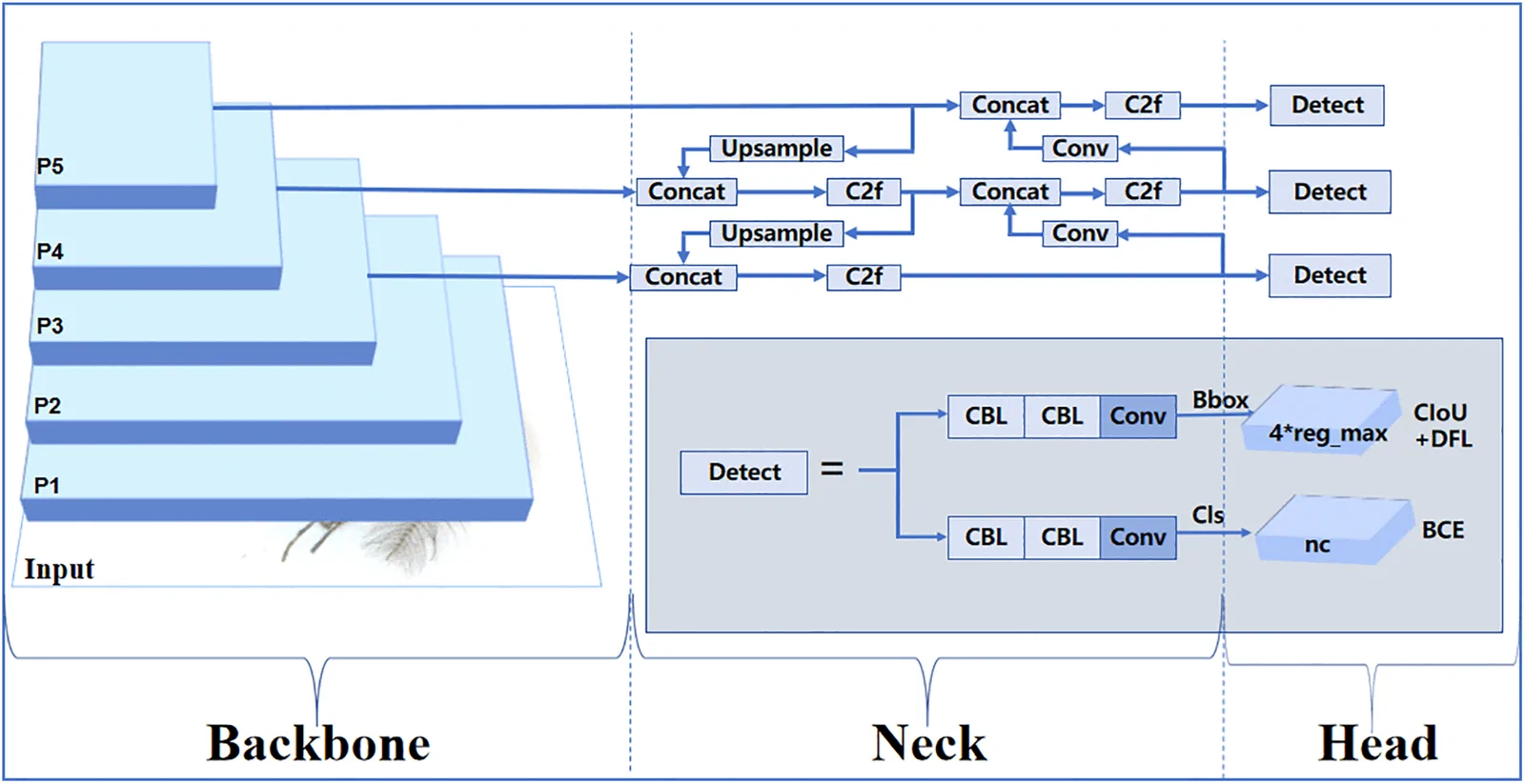
YOLOv8 architecture.

Integrating the strengths of prior YOLO versions, YOLOv8 retains lightweight features while boosting detection accuracy via optimized network design: it adopts the ELAN concept and C2f module, deploys a decoupled anchor-free detection head with advanced assignment and loss mechanisms, and refines data augmentation strategies. It offers multi-scale variants and supports instance segmentation, achieving an excellent balance between detection performance and computational efficiency. This makes it highly suitable for real-time applications on resource-constrained embedded and mobile platforms, and the YOLOv8n model was specifically used in our research.

### 2.3. Experimental setup

The mosquito detection algorithm in this study was implemented using the PyTorch framework, the experimental setup included Python 3.8, PyTorch 1.12.0, and CUDA 11.3. The hardware used for the experiments consisted of an NVIDIA GeForce GTX 1650 GPU, an Intel Core i5-9300H processor (2.40 GHz), and 16 GB of RAM (8×2 GB).

#### 2.3.1. Image preprocessing methods.

To enhance the robustness and generalization performance of the model while retaining the biological integrity of mosquito morphological features, a series of image-preprocessing and augmentation techniques were applied. The mosaic augmentation method was used to randomly concatenate multiple images, increasing data diversity without distorting key morphological characteristics. To simulate orientation variations caused by differences in specimen placement or camera angle during image acquisition, horizontal flipping was employed to improve the adaptability of the model to different mosquito postures. The image color space—hue, saturation, and brightness—was randomly adjusted within moderate ranges to simulate realistic variations in illumination and enhance the generalization ability of the model. The magnitude of these adjustments was constrained to avoid excessive color distortion or unrealistic changes in the original images. Geometric transformations such as translation and scaling were used to enhance robustness against positional and scale differences commonly observed during image capture. In these operations, the structural integrity of the specimens was preserved. Additionally, random erasing was introduced to simulate partial occlusions (e.g., foliage). All images were resized to 640×640 pixels to ensure input consistency. These augmentation strategies were employed to improve the detection accuracy and generalization of the model across diverse and complex scenarios.

#### 2.3.2. Hyperparameter settings.

Several key hyperparameters were used to optimize model training, as shown in Table 3. First, the learning rate (lr0) was set to 0.01 to ensure faster convergence during the initial stages of training. Weight decay was set to 0.0005 to control the complexity of the model and prevent overfitting. The momentum was set to 0.937 to accelerate training and improve model stability. To further enhance model performance, a three-round warmup strategy (warmup_epochs) was employed, set to 3.0, allowing the model to gradually adapt to the optimization target during the early training phases. To improve detection accuracy, the weight of the box loss function was set to 7.5, the weight of the class loss function (cls) was set to 0.5, and the weight for deep feature learning (dfl) was set to 1.5. A non-maximum suppression strategy was applied with an intersection over union (IoU) threshold of 0.7 to suppress overlapping bounding boxes and improve detection accuracy. By appropriately configuring these hyperparameters, model training effectiveness was optimized, leading to enhanced performance and generalization capability in object detection.

**Table 3:** Hyperparameter configuration.

Hyperparameter	Value	Description
Learning Rate (lr0)	0.01	Initial learning rate used for weight updates during training
Weight Decay (weight_decay)	0.0005	Controls model complexity and prevents overfitting
Momentum	0.937	Accelerates the training process and improves model stability
Warmup Epochs	3.0	Gradually adapts the optimization goal in the initial training phase
Box Loss Weight (box)	7.5	Weight of the loss function for the bounding box
Class Loss Weight (cls)	0.5	Weight of the loss function for class prediction
Deep Feature Learning Weight (dfl)	1.5	Weight of the loss function for deep feature learning
Non-Maximum Suppression (NMS)	True	Applies NMS to suppress redundant bounding boxes
IOU Threshold	0.7	IOU threshold for NMS, controls the selection of overlapping boxes

#### 2.3.3. Performance evaluation of deep-learning models.

The performance of the deep learning models was evaluated using a combination of confusion matrices and several quantitative indicators, including precision, recall, F1-score, mAP50, and mAP50-95. Precision represents the proportion of true positive samples among all predicted positive results generated by the model, and recall characterizes the model’s effectiveness in accurately recognizing all real positive instances in the dataset.The F1-score, defined as the harmonic mean of precision and recall, provides a balanced assessment of overall detection performance. In addition, mean Average Precision (mAP) was adopted as a key evaluation metric, where mAP50 corresponds to the average precision at an IoU threshold of 0.5, and mAP50-95 denotes the average precision calculated across multiple IoU thresholds ranging from 0.5 to 0.95.

To further examine the classification performance among different categories, confusion matrices were employed for inter-class comparison, thereby validating the reliability and accuracy of the model in both detection and classification tasks. Considering the requirement for model light-weighting, additional factors such as model parameter size, computational complexity, training efficiency, and inference speed were also taken into account. Collectively, these evaluation metrics provide a comprehensive and reliable assessment of the model's performance and robustness in various object detection scenarios. The mathematical formulations of several evaluation indicators are presented as follows:


$$\begin{array}{c} Precision=\frac{Tp}{Tp+Fp}, \end{array}$$ (1)

$$\begin{array}{c} Recall=\frac{Tp}{Tp+Fn}, \end{array}$$ (2)

$$\begin{array}{c} F1-Score=\frac{2*Precision*Recall}{Precision+Recall}, \end{array}$$ (3)

$$\begin{array}{c} mAP=\frac{\sum_{i=1}^{C}AP_{i}}{C}, \end{array}$$ (4)

where *Tp*, *Tn*, *Fp*, and *Fn* denote the number of true positives, true negatives, false positives, and false negatives, respectively; C represents the total number of categories, and APi denotes the average precision (AP) for the i-th category.

### 2.4. Deployment on mobile devices

#### 2.4.1. Mobile deployment framework.

In this study, the Neural Computing Neural Network (NCNN) framework was adopted to accelerate YOLOv8 model inference, and Android Studio served as the dedicated development environment for deploying the model on mobile platforms. The developed mobile application supports image import from the gallery for detection, along with real-time video and image detection functionalities, thereby enabling on-site real-time mosquito identification. For seamless deployment, NCNN was integrated with OpenCV-Mobile—responsible for image preprocessing and postprocessing—while NCNN-Android -Vulkan further boosted YOLOv8 inference efficiency. Android Studio was preconfigured with JDK, SDK, NDK, CMake and Gradle to build a complete development toolchain, ultimately delivering a practical and efficient mobile solution for on-site mosquito detection.

#### 2.4.2. Implementation.

Before deploying YOLOv8 on the mobile device, it is first converted to a format supported by NCNN. As the trained PyTorch model (.pt file) is not directly compatible with NCNN, it must first be converted into the ONNX format. Using Ultralytics version 8.3.9 with onnx, onnxruntime-gpu, and onnxslim for export and optimization, the ONNX model can be visualized via Netron. The ONNX model is then further converted into NCNN-readable parameter (.param) and binary (.bin) files, with FP16 quantization applied to enhance inference performance. The generated param and bin files are placed in the assets directory of the NCNN model, and input/output parameters, category names, and the number of categories are adjusted as required for correct inference. Finally, the mobile application is compiled and run via Android Studio [35,36]. The conversion process is shown in Fig 5.

**Fig 5:**
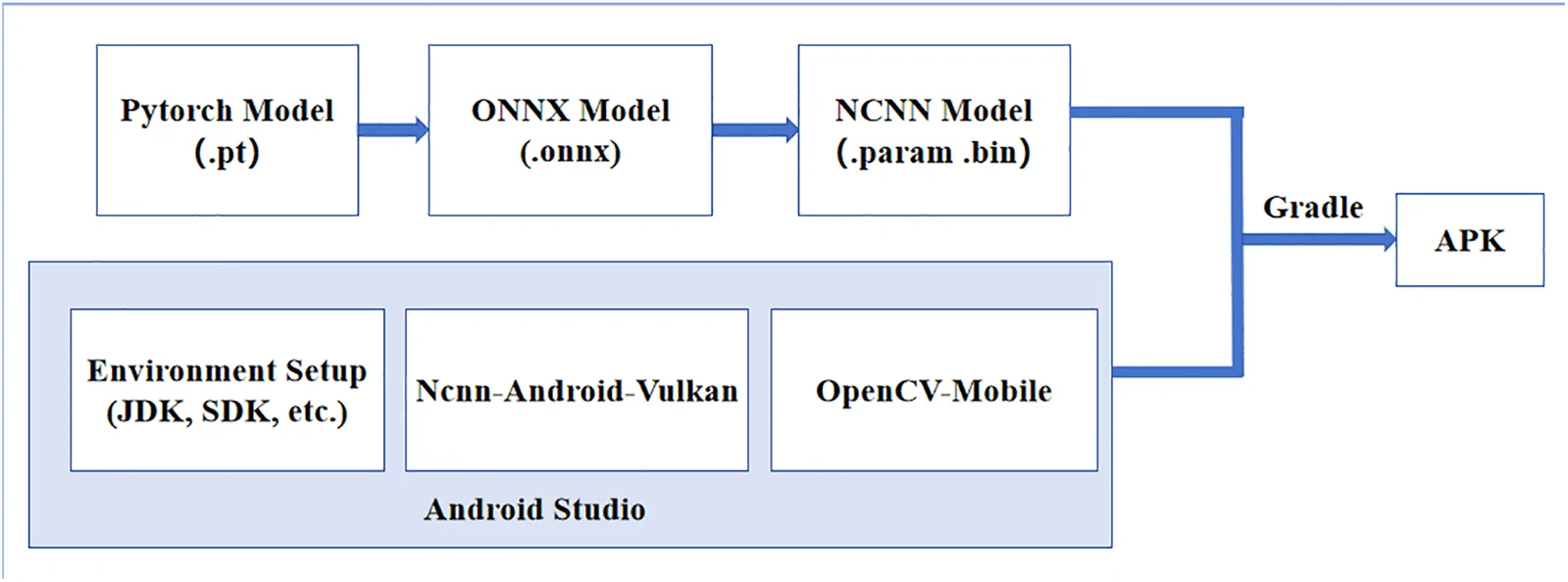
Flow chart of model transformation.

To implement the image and video detection functions on the mobile platform, a functional workflow was designed through collaboration between the Java and C layers. The image-detection function starts with the user inputting a Bitmap format image data through the Java layer. This data is passed to the C layer via a JNI interface, triggering the static image detection method for processing. In the C layer, the image data is converted into a suitable format (Mat) for further processing. Using the NCNN framework, deep-learning inference is performed with the pre-trained YOLOv8 model to detect targets and identify mosquito species in the image. After detection, the C layer returns the target box location and category in a HashMap format to the Java layer, which parses the results, draws the target box and label, and displays the detection results on the user interface. The video detection function follows a similar approach but adds the complexity of real-time video stream processing. The Java layer calls methods from the C layer via the JNI interface to activate the device camera, start the video stream, and configure the output window. The C layer continuously receives frames from the video stream, converts them to a suitable format (Mat), and uses the NCNN framework to detect objects. After processing each frame, the C layer overlays the target box and label onto the video frame, updating the display in real-time. Meanwhile, the Java layer manages the camera, video output window, and interactions with the C layer, ensuring immediate display of detection results. The detailed implementation of image and video detection is shown in Fig 6. The mobile deployment performance was evaluated on an Android smartphone equipped with the Qualcomm Snapdragon 8s Gen 3 system‑on‑chip. During real-time video detection, the system achieved an average processing speed of approximately 30 FPS, demonstrating its capability for real-time mosquito detection on mobile platforms.

**Fig 6:**
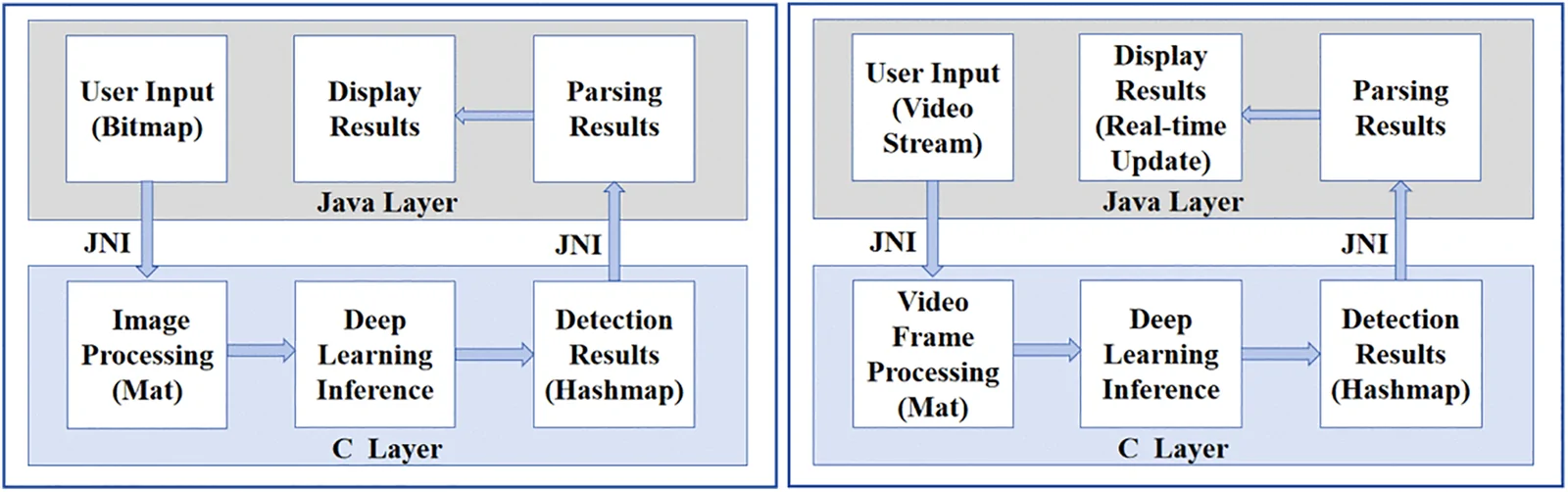
Implementation of image and video detection functions. (**a**) Flow chart of image detection. (**b**) Flow chart of video detection.

## 3. Results

### 3.1. Results of deep learning model training and evaluation

In the present research, we conducted a comparative analysis of multiple commonly used object detection algorithms for mosquito detection tasks, namely, Faster R-CNN (ResNet50), Faster R-CNN (MobileNetv2), YOLOv5, YOLOv8, and SSD. To comprehensively evaluate the performance of each algorithm, we compared multiple key metrics, as shown in Table 4. The quantitative results reported in Table 4 represent the average values obtained from multiple repeated measurements to reduce the influence of random variation. YOLOv8 achieves a precision of 0.988, a recall of 0.990 and an mAP of 0.992, all outperforming other algorithms. It also has lower computational cost and faster inference speed, making it well-suited for deployment on resource-constrained devices. Figs 7-9 show the detailed training results of YOLOv8, which further confirm its detection efficiency and accuracy. The performance metrics reported in Table 4 were calculated using a confidence threshold of 0.5.

**Table 4:** Comparison of performance across different models.

Model	Precision	Recall	mAP50	F1	Params (M)	FLOPs (B)	Inference Speed(ms)
YOLOv8	0.988	0.990	0.992	0.99	2.7	6.9	117
YOLOv5	0.982	0.983	0.989	0.98	1.8	4.3	154
SSD	0.746	0.790	0.985	0.77	13.8	15.4	143
MobileNet	0.871	0.665	0.933	0.75	82	42	435
ResNet50	0.831	0.789	0.960	0.81	42	135	459

**Fig 7:**
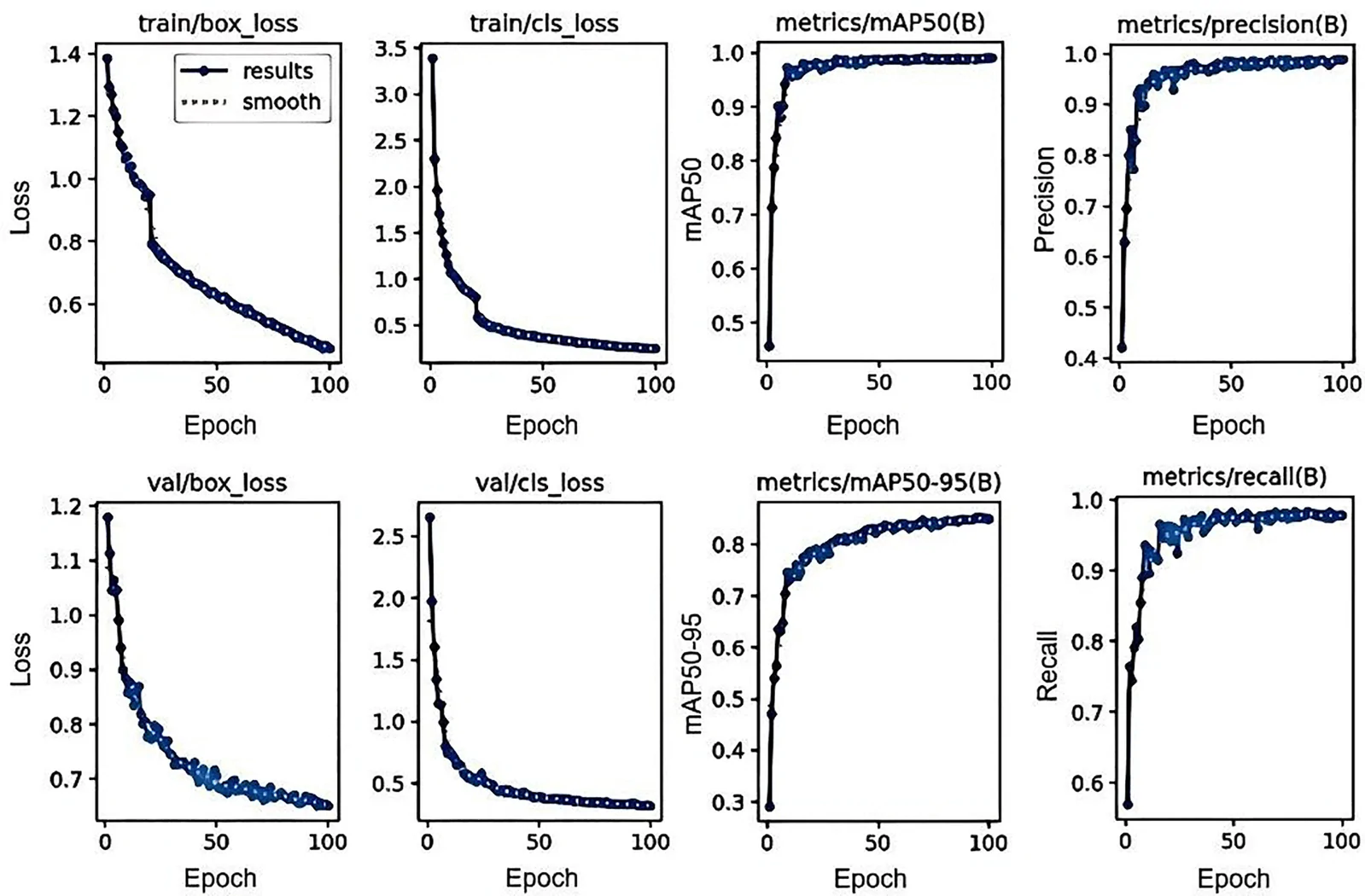
YOLOv8 training results (Loss, precision, recall, and mAP curves).

**Fig 8:**
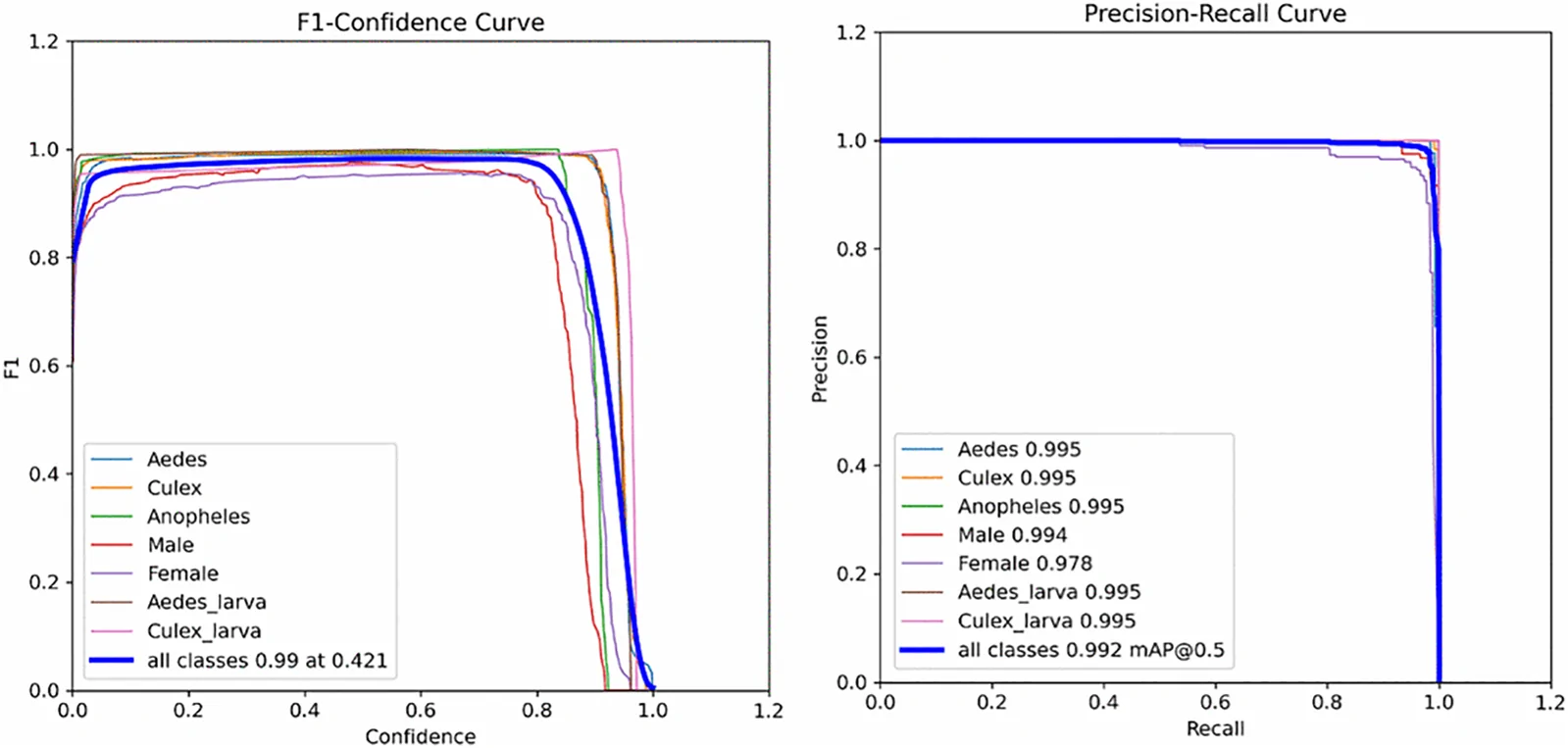
Precision-recall and F1-confidence curves of YOLOv8. (**a**) F1-Confidence Curve. (**b**) P-R Curve.

As shown in Fig 7, the box and classification losses for both training and validation datasets exhibit a consistent downward trend, indicating stable convergence without overfitting. The mAP at IoU=0.5 (mAP50) rapidly increases and stabilizes at 0.99, suggesting high detection accuracy. The mAP across multiple IoU thresholds (mAP50-95) also improves steadily, reaching 0.87, demonstrating model robustness in various detection scenarios. Furthermore, the precision and recall curves rapidly increase and eventually stabilize, indicating that the model rarely missed while maintaining a high detection rate.

As shown in Fig 8, the F1–Confidence curve (a) remains high and stable within the 0.1–0.8 confidence range, indicating that the model maintains a well-balanced trade-off between precision and recall across a broad confidence interval. This stability suggests that the performance of the model is not highly sensitive to confidence threshold variations, ensuring consistent detection reliability. The Precision–Recall (P–R) curve (b) encloses a large area, demonstrating that the model sustains high precision across different recall levels, which correlates with high mAP. Most classes exhibit nearly optimal precision–recall trade-offs, with curves closely approaching the (1.0,1.0) point, signifying strong detection capability. However, the Female category shows a slight drop in precision at higher recall values, suggesting a relatively higher false positive rate. This indicates potential room for improvement in handling this category. Overall, YOLOv8 achieved excellent detection performance with high precision, recall stability, and strong generalizability.

Fig 9 presents the confusion matrix, which reflects the classification performance of YOLOv8 across different categories: high classification accuracy for most classes, with *Aedes* (0.99), *Culex* (0.98), *Anopheles* (1.00), Male (0.97), Female (0.97), *Aedes*-larva (1.00), and *Culex*-larva (1.00), demonstrating that the model can effectively distinguish mosquito species. A small background portion is misclassified as *Culex*-larva (55%) and Female (30%), indicating that the model occasionally generates false positives in complex environments. A minor overlap (2%) between Female and Male is likely due to their morphological similarities. Despite these minor misclassifications, the model maintains strong overall reliability. Future improvements can be achieved by expanding the dataset, enhancing data augmentation methods, and refining the model architecture to further optimize training performance.

### 3.2. Display of mobile application results

When using the mobile app to detect mosquitoes, if the image contains the target, the app generates a prediction box and displays the predicted class and probability. In image detection mode, the user selects an image from the gallery by clicking "Select Image" and "Recognize" to display the corresponding prediction box and information. In video detection mode, by clicking "Video Recognition," the app activates the camera for real-time detection. If the target is detected, the prediction box and information are displayed on the video frame. Fig 10 shows the mobile detection results. This demonstrates that the mobile app effectively identifies mosquitoes, making mosquito detection practical for real-world applications.

**Fig 9:**
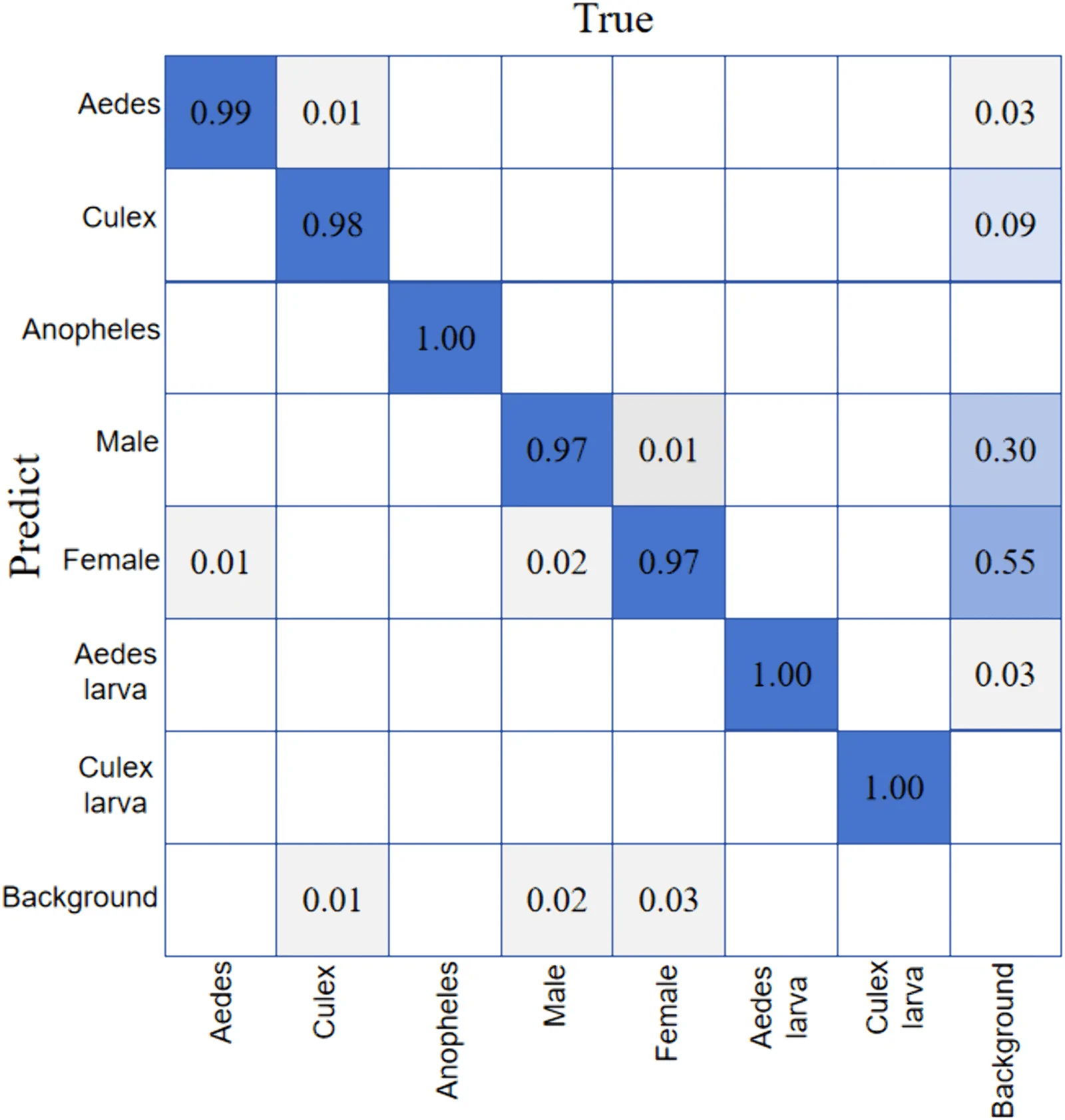
Confusion matrix.

**Fig 10:**
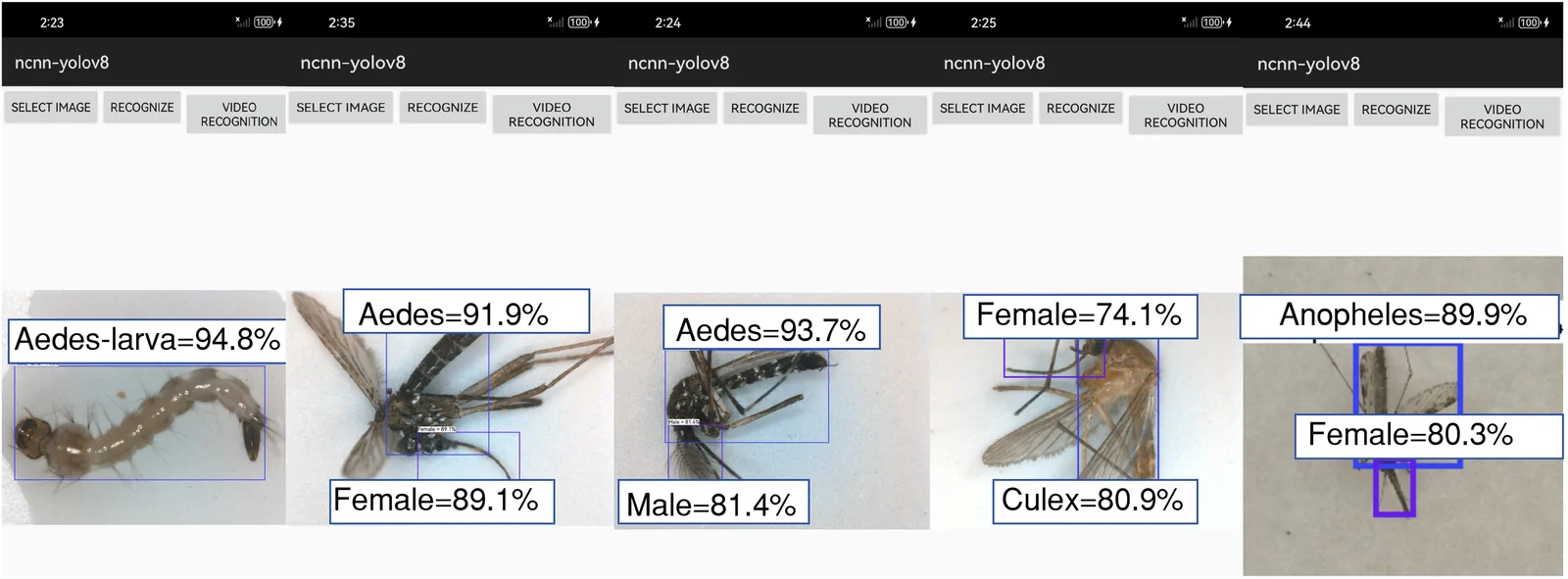
Result of mobile application.

## 4. Discussion

This study comprehensively evaluated five object detection algorithms (YOLOv8, YOLOv5, SSD, Faster R-CNN with MobileNet, and Faster R-CNN with ResNet50), using precision, recall, mAP50, mAP50-95, F1 score, model parameters, FLOPs, training speed, and inference speed as evaluation metrics. The results indicated that the comprehensive performance of the YOLOv8 algorithm was significantly superior to that of the other four algorithms, with its mAP50, mAP50-95, and F1 score reaching 0.992, 0.87, and 0.99, respectively. It exhibited balanced precision-recall characteristics and favorable model robustness. Additionally, the algorithm possessed both excellent running speed (training speed: 153 s/epoch; inference speed: 117 ms) and lightweight advantages (2.7M parameters, 6.9B FLOPs), which could effectively meet the real-time deployment requirements of resource-constrained devices.

It should be noted that this study still has certain limitations. In complex scenes, the model suffers from false positive detection issues: 55% of background regions were misclassified as *Culex*-larva, and 30% as Female mosquitoes, which may lead to overestimation of mosquito population size. Affected by morphological similarity and imaging conditions, the misclassification rate between female and male mosquitoes was 2%. Although male mosquitoes were relatively underrepresented in the dataset, they generally exhibit more distinctive sex-related morphological characteristics, particularly their strongly plumose antennae, which may provide more discriminative visual features for the model. In contrast, the sex-specific morphological features of female mosquitoes are relatively less prominent and may be more susceptible to variations in specimen posture, image quality, and partial occlusion. Therefore, differences in morphological feature discriminability, rather than sample size alone, may partly explain the slightly lower performance observed for female mosquitoes. Given that only Female mosquitoes are capable of transmitting diseases, such gender misclassification may interfere with the accuracy of vector population assessment, thereby resulting in deviations in public health intervention strategies. Furthermore, the model fails to effectively distinguish non-mosquito insects (e.g., Chironomidae, Dixidae, Trichoceridae, and Anisopodidae) from target mosquitoes, which is likely to increase the probability of false positive detection in natural environments.

The field application performance of the model is constrained by factors such as extreme lighting (e.g., overexposure, shadow occlusion), cluttered backgrounds, insufficient imaging resolution of mobile phone cameras, and motion blur. Future research may expand the dataset to include diverse samples such as non-mosquito insects and complex backgrounds, while enhancing the model’s adaptability to environmental variables to improve its robustness. Despite the aforementioned limitations, this study still provides important support for the development of automated portable mosquito monitoring systems and lays a theoretical and practical foundation for optimizing vector control programs.

## 5. Conclusions

This study adopts the YOLOv8 object-detection algorithm to develop an efficient mosquito-detection method. After training with a self-built dataset, the method can accurately distinguish adult forms of three mosquito species as well as larval forms of *Aedes* and *Culex* mosquitoes. Experimental results demonstrate that YOLOv8 excels in precision, recall, and mAP, achieving 0.988, 0.990, and 0.992, respectively, and an F1 score of 0.99 in mosquito species detection. In terms of lightweight performance, the model exhibits excellent results in parameters, computational load, and inference speed,consequently allowing it to be effectively deployed on devices with constrained resource capacities.By deploying the model on mobile platforms, the study enables mosquito detection on static images and supports real-time video stream detection, greatly enhancing detection efficiency and convenience. The successful application of this method provides an innovative and practical technical solution for the prevention and control of mosquito-borne diseases, enabling rapid and accurate identification of mosquito species.
